# A *WISER* way for simulating the performance of gratings

**DOI:** 10.1107/S1600577522009134

**Published:** 2022-10-18

**Authors:** Michele Manfredda, Lorenzo Raimondi, Daniele Cocco

**Affiliations:** a Elettra-Sincrotron Trieste SCpA, SS 14 Km 163.5, Basovizza 34149, Italy; b Lawrence Berkeley National Laboratory, 1 Cyclotron Road, Berkeley, CA 94720, USA; Uppsala University, Sweden

**Keywords:** wavefront propagation, diffraction gratings, coherent mode propagation, groove placing error

## Abstract

Soft X-ray monochromators for synchrotron radiation sources have been continuously developed over the years, improving energy resolution and stability. Great effort has been made in improving the surface quality of the optics involved, reaching values permitting diffraction-limited images. Still, one problem has not been solved, nor fully understood, yet: groove placing errors. Nowadays, these are one of the major factors responsible for resolving the power reduction of diffraction-gratings-based X-ray monochromators. Despite decades of use of gratings, there is not yet a universally established method for predicting and simulating the effect of groove placing errors on monochromator performance. This is especially important in the new scenario of high-coherent X-ray sources, *i.e.* diffraction-limited storage rings and free-electron lasers. To address this problem, in this article an approach based on *WISER* (*Wavefront propagatIon Simulation codE libRary*) is presented. *WISER* is a physical optics simulation package, also available in the user-friendly *Orange Synchrotron Radiation Suite* – *OASYS*. Even though it was originally conceived to assess the focusing performance of X-ray mirrors in the presence of height defects, it perfectly simulates the performance of a periodic (or quasi-periodic) structure like a diffraction grating. In this article, the way to use *WISER* and its application to a specific case, *e.g.* the design of a monochromator for the upgrade of a beamline at the Advanced Light Source, are shown. A simple rule for estimating how well the grooves are placed on a grating, based on calculation of the Strehl ratio, is also presented.

## Abstract 2

The groove placing errors of X-ray diffraction gratings can be precisely simulated by using a physical optics simulation package. How the groove placing errors influence the energy resolution and the spot profile will be shown. An empirical formula to estimate the precision of manufacturing of the grating is also derived.

## 1. Introduction

Diffraction gratings have been used extensively in synchrotron radiation facilities for several decades and, more recently, in free-electron lasers. But the history of using gratings can be tracked back to 1786. This is the year David Crockett was born, but it was a different David who made gratings a scientific tool in that year, *e.g.* the astronomer David Rittenhouse. And the principle of diffraction gratings is even antecedent to this date, some time in the late seventeenth century (by James Gregory).

Diffraction gratings have been, indeed, valuable instruments in science, especially in astronomy, and, as with most of the instrumentation used in science, have been subject to improvement, starting from their ability to separate wavelengths. Their applications range from astronomy to nano-scale microscopy and from the deep infrared to tender X-rays (or from micrometres to angstroms in wavelength).

The gratings used in the synchrotron radiation scientific community are almost always unique pieces custom-made for a specific application. They can be commercially available, either produced holographically or through mechanical ruling machines. In an attempt to overcome some of the limitations of the commercially available gratings, some laboratories have developed, or are developing, in-house facilities to produce gratings targeted to specific applications (*e.g.* Siewert *et al.*, 2018 ▸; Voronov *et al.*, 2015 ▸, 2019 ▸). Improvement in the quality of the gratings has enabled the achievement of higher and higher energy resolution thanks to the ability to place every single line of the grating closer and closer to its ideal location. Metrology techniques have been developed to characterize the groove-placing precision (Irick & McKinney, 1997 ▸; Cocco *et al.*, 2003 ▸; Gleason *et al.*, 2017 ▸), and help to predict the performance of beamlines.

To simulate the effect and behavior of the diffraction gratings, the most common method has been (and still is) the use of ray-tracing programs. The most widely used (by the synchrotron radiation community) was developed in the 1980s at University of Wisconsin, Madison, USA, by a team led by Franco Cerrina (Lai & Cerrina, 1986 ▸). Several subsequent improvements and advanced visual user interfaces have been made along the years to address the need of the opticians. The most recent, and most complete, is its implementation in *OASYS* (*OrAnge SYnchrotron Suite*). *OASYS* is a graphical environment gathering most of the simulation tools that have been developed, and used, within the X-ray optical community (Rebuffi & Sanchez del Rio, 2017 ▸; Shi *et al.*, 2014 ▸).

**Figure 1:**
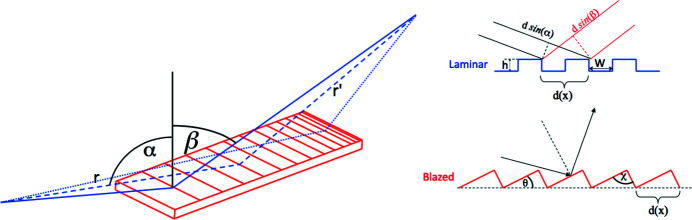
Left: convention used for the angles of incidence and diffraction. Right: pictorial description of the laminar and blazed gratings with relative nomenclature. *d*(*x*) is the period. α and β are the angles of incidence and diffraction, respectively, considered positive with respect to the normal. *h* is the groove depth for laminar gratings and *W* is the groove width. θ is the blaze angle and χ the apex angle. *r* is the source-to-grating distance and *r*′ is the grating-to-focus distance.

In ray-tracing simulations, each photon is propagated as an independent ray, whose trajectory modification depends on the local characteristics of the particular optical element it impinges on. In the case of a diffraction grating (see Fig. 1 ▸ for conventions) the relation between the incoming and diffracted beam directions of a photon of wavelength λ, interacting with the grating at the location (*x*, *y*), is calculated from the following relation,

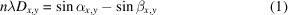

where *D*_*x*,*y*_ is the groove density of the grating at the location *x*, *y*, and *n* is the diffraction order (α and β are the local incident and diffraction angle as represented in Fig. 1 ▸).

Since, in a standard diffraction grating, the groove density is important only in the tangential (or meridional) direction, for the sake of the discussion in this article we will consider only the dependence of *D* from the coordinate *x*, along the grating meridional plane. The groove density *D*_*x*_ can be chosen to be constant, along the grating, or variable according to a defined polynomial law in the form

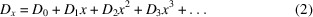

where *x* = 0 coincides with the center of the grating. In a ray-tracing program, equation (2) is used to calculate the local groove density at a given location and then equation (1) is used to define the new direction of propagation of that particular ray or photon.

As much as ray tracing is able to predict reasonably well the resolving power of an X-ray monochromator, with the photons treated purely geometrically, it fails in properly assessing the effect of groove placing errors and on the diffractive effects due to the coherent length of the beam over the grating surface. Moreover, to the best of our knowledge, no ray-tracing program, freely available to the public, is designed to introduce an arbitrary groove density distribution, either the measured one or an artificial one different from a polynomial distribution with a limited number of terms. This is, indeed, something easily solvable, but, still, something to be taken into account.

A second limitation, related to the first one and usually not important in standard synchrotron beamlines, is the insensitivity to the number of illuminated lines, *N*. Practically, it does not matter how many grooves are illuminated by the radiation, or how long the coherence length of the beam over the grating is. A ray-tracing program can estimate a resolving power *E*/Δ*E* (or λ/Δλ) in excess of, for instance, 20000 even if only 5000 lines are coherently illuminated. This is, of course, not physically possible since the maximum resolution achievable cannot overcome

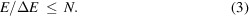

Of course, even if ray-tracing has all the above-mentioned limitations and cannot provide directly the wavefront or calculate the coherent effect, it is still a very powerful method. In particular, its ability to easily handle multi-wavelength (or continuous energy) sources permits simulating the bandwidth selected by a monochromator, or any of the chromatic aberrations introduced by the optics of a beamline.

To overcome the limitations of ray-tracing, wavefront propagation tools have been developed, aimed at addressing applications related to the diffraction-limited storage rings (Shi *et al.*, 2014 ▸; Chubar & Elleaume, 1998 ▸; Raimondi *et al.*. 2013 ▸), to astrophysics (Raimondi & Spiga, 2015 ▸), and to highly monochromatic and coherent sources such as free-electron lasers [see, for instance, *WISER* (*Wavefront propagatIon Simulation codE libRary*) (Manfredda *et al.*, 2020 ▸)].

*WISER* is an evolution of *WISE*, originally aimed at stimulating the point spread function of X-ray telescopes (Raimondi & Spiga, 2015 ▸), and it will be the protagonist of this paper. Except where explicitly stated, all the simulations shown in this paper have been performed using *WISER*.

## 2. WISER

### 2.1. Scope and design

**Figure 2:**
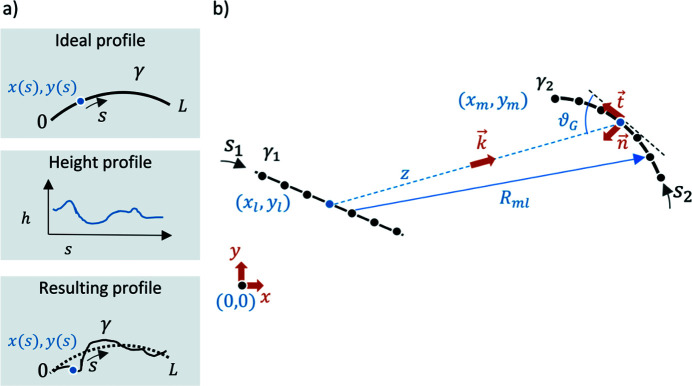
(*a*) Top: an ideal surface profile γ representing an optical element. Middle: height profile as a function of *s*. Bottom: the summation of the two. (*b*) The propagation from γ_1_ to γ_2_. The output wavevector **k** is defined by the reflection on the ideal profile, without considering any height profile superimposed on it.

*WISER* is a physical optics simulation software designed to propagate a wavefront along a train of optics. Its method is based on the application of the Huygens–Fresnel principle on the grazing-incidence reflection. This allows working in Kirchhoff’s scalar approximation, assuming spatially coherent and monochromatic radiation. The aim is the assessment of the focusing performance in the presence of surface error defects and of other geometrical deviations from the nominal configuration (such as misalignments, *etc*.). The surface error is modeled following the traditional distinction between figure error and roughness. The first one is provided as a residual height profile with respect to the ideal surface and the second one is provided as the power spectral density of the surface height profile. *WISER* is specialized in the spectral range from the extreme ultraviolet to hard X-rays, thus typically operating at grazing angles of incidence, where the reflectivity is higher. At grazing angles of reflection/diffraction the effect in the incidence plane is much larger than in the transverse one by a factor equal to one over the cosines of the angle of incidence. The sagittal error of the optical surfaces is therefore neglected, and only the longitudinal profile is considered. For this reason, an optical element, in *WISER*, is represented by its (median) tangential profile, which is a 1D curve (γ), parametrized as γ → {*x*(*s*), *y*(*s*)} in a 2D Cartesian reference frame (*X*, *Y*) (Fig. 2 ▸). For instance, ellipsoidal mirrors are represented by elliptic arc sections, paraboloid mirrors by parabolic arc sections, plane mirrors and detectors by line segments, and so on. The complete mathematical description of the method can be found in Raimondi & Spiga (2015 ▸).

The ideal mirror profile γ(*s*) can be modified by adding a custom height profile *h*(*s*), where *s* is a spatial variable in the tangential direction of the surface [Fig. 2 ▸(*a*)]. Such a feature is primarily used to input the surface error profiles, but it also enables the modeling of a complete new optical profile; or, as described later, to add the grating’s groove profile to the existing *WISER* optical surface, as if it were a ‘surface error’. In doing so, the orientation of the wavevector **k**_out_ leaving the (modified) optical element is still computed based on the original shape. A manual correction is necessary in case the ‘new’ emerging direction varies considerably. This is exactly what happens when the height profile is modeled to mimic the behavior of a grating. This approach will be discussed in the following sections.

*WISER* is written in Python (version 3.5 or higher) and consists of two distinct packages: *LibWiser* 1, which implements the computation library, and *OasysWiser* 2, which provides the graphical interface for *OASYS* (*OASYS* add-on). *LibWiser* contains all the code necessary for the simulations, such as the element collection, the positioning engine, the propagation manager, and the low-level propagation algorithms. It embraces all the *WISER* functionalities, and it can be used as a self-standing Python package by users with advanced programming experience. Conversely, *OasysWiser* – also referred to as the *WISER* graphic user interface (GUI) – is designed to cover the most common cases of use, and it is accessible to most users: it does not require any programming experience, and the system’s parameters (such as source wavelength, optical element length, propagation distances, gracing angles) are entered via GUI. This paper considers the latter case only, as described in Section 2.4. *WISER* runs on any machine equipped with Python 3.5. The *Numba* package provides just-in-time compilation to improve the speed in the computation of Huygens–Fresnel integrals (see Section 2.2), automatically parallelizing the computation across the available CPU-logical processors. In its present version, *WISER* does not take advantage of graphic processor unit (GPU) computing. The computation time grows approximately quadratically as a function of the number of samples. As an example, on an Intel Core i7-7700HQ CPU at 2.8 GHz (four cores, eight logical processors), typical computation times are: *t*_c_ ≃ 3 s for 10^4^ samples, *t*_c_ ≃ 55 s for 5 × 10^4^ samples, *t*_c_ ≃ 200 s for 10^5^ samples.

### 2.2. Numerical field propagation in *WISER*

The field *E* propagates from the source element γ_1_ to the target element γ_2_ [Fig. 2 ▸(*b*)]. The Huygens–Fresnel integral can be written as

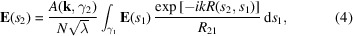

where λ is the wavelength, *k* = 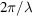 is the wavenumber, **E** is the complex-valued field and *R*_21_ is the scalar-value distance running from *s*_1_ to *s*_2_,

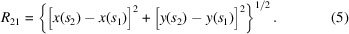




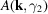
 is an effective aperture factor weighting the total amount of light collected by the optics. According to Raimondi & Spiga (2015 ▸), *A* approximates the cross section of γ_2_ seen from **k** whenever the local divergence of the beam, on the arrival optics, is much smaller than the grazing angle ϑ_G_. If 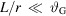 , where *r* is the average curvature radius of the wavefront and *L* is the size of the optical element, then *A* ≃ 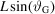 . Numerically, the integral of equation (5) is evaluated by means of an explicit recursive summation of the complex exponentials in the form

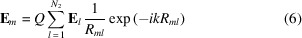

and

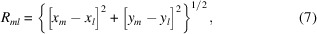

where *l* runs over the source sample grid and *m* runs over the destination sample grid.

When the first element of the chain (*i.e.* the ‘light source’) can be modeled analytically, the propagation on the following element is computed with the respective analytical expression rather than through equations (4)–(6). This happens, for instance, when point-like emitters and Gaussian sources are used.

As a second remark, we observe that the fast Fourier transform (FFT) algorithm is not used contrary to other common methods for wave propagation. The FFT is effective for propagation between parallel planes. Still, it introduces approximations on the phase when highly tilted planes are involved, which is precisely the case of grazing-incidence X-ray optics. Generally speaking, the numerical propagation between tilted planes is of particular interest, as shown by Stock *et al.* (2017 ▸) and references therein. To the authors’ knowledge, a quantitative comparison between the two approaches for grazing-incidence optics affected by surface error has not been done yet and would be an exciting topic for future investigations.

### 2.3. Sampling

**Figure 3:**
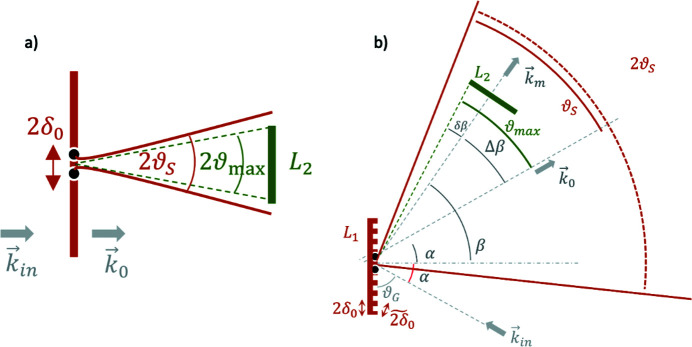
Diffraction-based models for finding the grid spacing δ_0_. (*a*) The angle in transmission geometry, and (*b*) diffraction from a (reflection) grating of radiation impinging along **k**_in_ and exiting along **k**_m_. 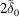 is the slit width seen by the incident radiation.

In order to ensure an accurate simulation, the choice of the proper grid spacing (δ_0_) is critical. A limit on the maximum spacing is imposed by means of the Nyquist criterion, whose most common formulation states that the spacing δ_0_ must be chosen such that 2δ_0_ ≤ *f*_max_, where *f*_max_ is the maximum spatial frequency of interest. In the case of interest here, the maximum frequency is better found by reformulating the problem in terms of angular bandwidth. Fig. 3 ▸(*a*) shows the simplified case where the source and detector planes are parallel and the radiation propagates normally with wavevector **k**_0_. The elementary 2δ_0_-wide extension on the source (corresponding to three point-wise emitters) produces a diffraction pattern that can be likened to that of a 2δ_0_-wide slit, which diffracts most of the total energy within the half-angle ϑ_*D*_ = λ/2δ_0_. To properly illuminate the arrival plane, the diffraction angle ϑ_*D*_ must be greater than the angle subtended by the arrival plane itself, which represents the maximum angle of interest of the system ϑ_max_. By approximating 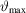 ≃ 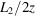 (with *z* being the distance between the planes) and by having 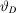 ≳ 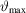 one obtains 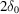 ≲ 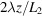 , where the right-hand term denotes the maximum frequency *f*_max_. In the case of a grating [Fig. 3 ▸(*b*)] the former reasoning still holds, provided that ϑ_*D*_ and ϑ_max_ are properly re-defined. The characteristic diffraction angle is now 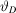 = 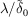 , where 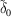 = 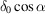 is the reduced size of δ_0_ as seen by a plane wave impinging on the grating with incidence angle α [Fig. 3 ▸(*b*)]. Similarly, the maximum angle is now computed as ϑ_max_ = β − α + δβ, where β − α is the deviation angle of the *m*th order with respect to the zero order and δβ ≃ *L*_2_/2*z* accounts for the angular size of the detector. Ultimately, by constraining 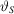 ≳ 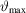 , one has

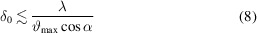

where

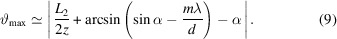




### 2.4. Simulating a diffraction grating with *WISER*

At the time of the simulations reported in this paper, *WISER* did not yet offer an optical element implementing the behavior of a grating. For this reason, a workaround was used instead of the best practice solution. Since the workaround is as creative and useful as the best practice, here we will briefly sum up both of them. In addition, whereas best practice requires specific programming skills to modify the libraries, the workaround is also accessible to the average user, via graphic interfaces.

#### 2.4.1. The best practice

The best practice for introducing a new optical element requires implementing two functions in the code: (1) the function 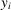 = 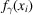 that returns the optical profile γ = {*x*_*i*_, *y*_*i*_} for a set of samples *x*_*i*_; and (2) the function returning the direction of the output wavevector **k**_out_. The former enables computing the field in the whole space as explained in Section 2.2, making the algorithm properly place the subsequent optical element along the propagation direction. In the case of elements that can possibly deliver radiation in many directions (as for a grating), a unique propagation direction must be specified (*e.g.* by selecting the desired diffraction order). Notice that, since *WISER* propagates the field in all the possible directions, any element arbitrarily placed in the space will collect some kind of light. But only those elements that are placed along the propagation direction will return meaningful (*i.e.* non-noise-like) intensity distributions.

#### 2.4.2. The workaround

**Figure 4:**
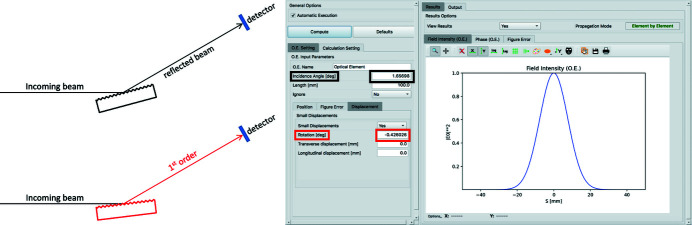
The ‘workaround’ process starts by defining the proper position/direction of the beam and the detector by ‘telling’ *WISER* that the element is a mirror (top left image), and setting the angle of incidence (black rectangles in the right image). Then a rotation is applied to the optical element (red rectangles in the right image) to have the required angle of incidence on the grating to diffract the beam in the detector direction (lower left image).

Within the *OASYS* interface, the same task described above can be achieved by providing a convenient height profile *h*(*s*) that includes, together with the surface defects (if needed), any departure from the available mirror profiles including the desired optical surface of a grating (*e.g.* of each groove along the grating). However, in doing so, the direction of the exit wavevector **k**_out_ still remains the same as of the primitive surface (*e.g.* a mirror), even if the new height profile changes the propagation direction by an angle that we will call Δφ. This effect can be compensated by means of the ‘Rotation’ parameter, available in *WISER*, which applies an arbitrary rotation to the optical geometry without altering the output wavevector direction. Let us consider a grating for which the angle of incidence is α_G_ and the angle of diffraction is α_G_ + Δφ = β_G_, so that the total angle of deviation from the incidence beam to the diffracted beam is α_G_ + β_G_. To center the detector in the correct direction, we need to tell *WISER* that the grating is at an angle half way between the incident and the diffracted direction, *e.g.* (α_G_ + β_G_)/2, that is equal to α_G_ + Δφ/2. But we want to have the beam impinging on the grating with the correct angle, so we need to use the ‘Rotation’ parameter to tilt the mirror back by Δφ/2 so that the actual angle of incidence is α_G_. Fig. 4 ▸ shows the panel in *WISER* on *OASYS* where the ‘Rotation’ can be inserted, and also helps in understanding the logic behind those rotations.

Ultimately, a convenient recipe to emulate a diffraction grating is: (*a*) start from a plane mirror; (*b*) set the mirror height profile *h*(*s*) equal to the required groove profile *h*_G_(*s*) (plus any profile deviation from a flat surface); (*c*) set the incidence angle equal to (α + β)/2; (*d*) apply a rotation of (β − α)/2.

#### 2.4.3. Generation of the profile and effect on the reflected beam

To generate the grating profile, it is necessary to calculate the location of each groove on the grating surface. The sequential number of a given line located at a distance *L* from the grating center can be obtained by integrating equation (2) as

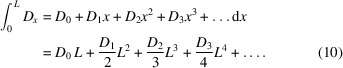

By using equation (10), one can generate either a laminar or a blazed profile for the grating by simply adding the proper geometrical profile of the grating’s groove to each line.

**Figure 5:**
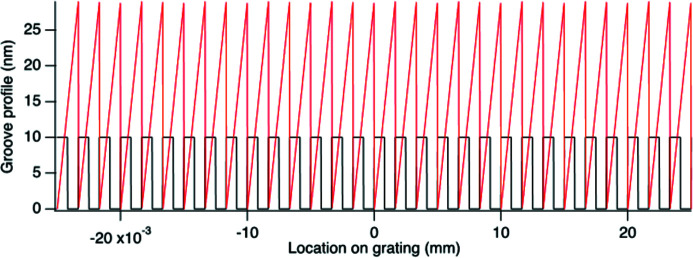
Groove profiles in the center of the grating with *D*_0_ = 600 l/mm, *D*_1_ = 0.4 l/mm^2^ and *D*_2_ = 4 × 10^−5^ l/mm^3^. The black curve is for a laminar grating with 10 nm depth. The red curve is for a blazed grating with a 1° blaze angle.

An example is shown in Fig. 5 ▸, where the profile of a 600 lines mm^−1^ (l/mm) grating is shown for a laminar grating with 10 nm depth (black profile) and a blazed grating with 1° blaze angle (red profile).

Before using the distribution of the lines to diffract and monochromatize the light, let us consider one more thing that *WISER* can provide that ray-tracing cannot, *e.g.* the effect of the profile of the grooves on the reflected beam (*e.g.* with the grating used in zero-order). When the beam is simply reflected by the grating, the profile of the grooves is seen, by the beam, as a shape error. In most cases, what happens to the zero order is not important. The gratings are usually optimized to deliver the maximum efficiency in the first order and all efforts are made to optimize the monochromatic beam efficiency and dimension. But, for instance in a free-electron laser, where the radiation is already relatively monochromatic, the zero order can be used to deliver an intense beam to the experimental station. A more sophisticated use of the zero order is described by Svetina *et al.* (2016 ▸), where the first order is diffracted and focused to an on-line ‘non-invasive’ energy spectrometer and the zero order to the experimental station.

In the presence of a coherent source, the quality of the focused spot can be predicted by calculating the Strehl ratio *S* (Strehl, 1895 ▸). The Strehl ratio *S* is the ratio of the actual peak image intensity compared with the maximum intensity if using a perfect optical system. It can be calculated as

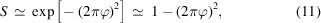

where φ is the phase error (with respect to the ideal case) depending on the angle of incidence θ and induced by the r.m.s. shape errors δ*h* through the following equation,

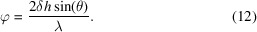

**Figure 6:**
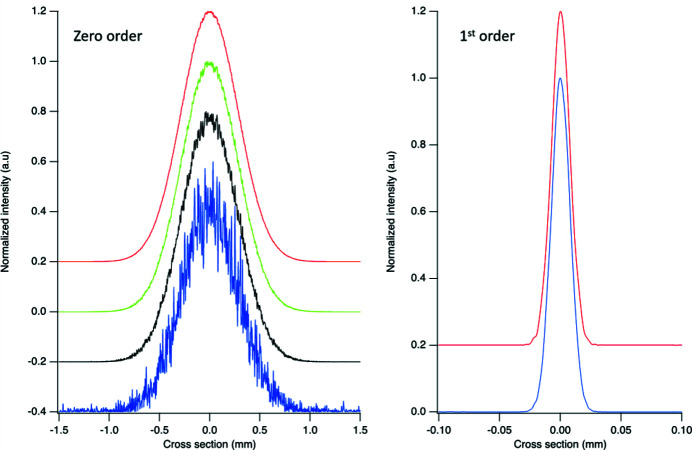
Cross section of the unfocused zero order (left) and focused first order (right) at 920 eV. The groove depths are 1 nm (red curves), 2.5 nm (green), 5 nm (black) and 10 nm (blue). Because there is no appreciable effect on the focused first-order beam, only the profiles for the extreme cases are shown.

Let us consider an example using the 600 l/mm grating whose profile was shown in Fig. 5 ▸. This is a variable-line-space grating with *D*_0_ = 600 l/mm, *D*_1_ = 0.4 l/mm^2^ and *D*_2_ = 4 × 10^−5^ l/mm^3^. The grating profile is laminar and the groove depth, in the simulation, is varied from 1 to 10 nm. The source-to-grating distance is 20 m and the focal distance of the first order is 5.1 m. The result of the simulation is shown in Fig. 6 ▸. The profile of the first order is not affected by the depth of the groove (lower right image in Fig. 6 ▸). This is due to the fact that each groove introduces a designed phase shift equal to one wavelength, but not a ‘random’ phase error. The reflected zero order, instead, is affected by the groove depth. Increasing the depth of the grooves, the out-of-focus intensity of the beam departs more and more from the ideal Gaussian profile. The grooves are seen by the reflected beam as a very high frequency shape error, inducing intensity oscillation at high frequency rather than a smooth deviation from a Gaussian profile. The departure from the ideal Gaussian profile is shown in the top right part of Fig. 6 ▸.

The Strehl ratio has a physical meaning only if the beam is focused. This is not the case of the image shown in Fig. 6 ▸. But, as already adopted in the case of the mirrors, one can use the phase error to estimate the departure from an ideal Gaussian profile of a beam out of focus. For the example shown here, where the angle of incidence is 1.7° and the photon energy 920 eV, *S* varies from 0.967 with 1 nm groove depth to ≪0.1 with the depth as high as 10 nm. A Strehl ratio around, or better than, 0.8 (coincident here to a 2.5 nm depth, or the black curve in Fig. 6 ▸) is likely desired to minimize the deviation from a Gaussian profile.

## 3. Effect of the groove placing error

The groove positions calculated with equation (10) are for a perfectly produced grating. However, it is always possible to add an error to the local position of each groove. This permits the generation of a more realistic grating. This is, in practice, equivalent to adding a slope (or shape) error to a perfect mirror. To the groove location calculated with equation (10), one can add an arbitrary function, simulating either a statistical error or a periodic (or the sum of some characteristic periodic) misplacement. The local difference between where a given line should be and where it actually is, is called the groove placing error. Together with the non-corrected aberration of the optical system, the groove placing error determines the quality of the spot focused by the grating.

The question we want to answer now, with the help of *WISER*, is: what groove placing error can we tolerate before it affects either the resolution or the flux? An initial answer to this question has been proposed by Cocco & Spiga (2019 ▸) and Gleason *et al.* (2017 ▸).

In Cocco & Spiga (2019 ▸), the equation for the Strehl ratio of an arbitrary diffraction order from a grating has been derived starting from the r.m.s. groove placing error δ as

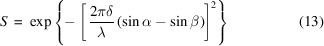

(with the convention for α and β as defined in Fig. 1 ▸). This equation affects only the diffracted beam, not the reflected one for which α = β, so *S* is equal to 1. From equation (1), we know that 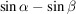 is equal to *n*λ*D* or *n*λ/*d* (*d* is 1/*D* corresponding to the groove width or *d*-spacing). Consequently, equation (13) can be rewritten as

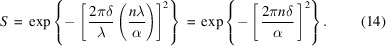

Surprisingly enough, the Strehl ratio depends only on the groove density (or *d*-spacing) and not on the photon energy or angle of incidence. As simple as it is, this equation is still a valid guideline to estimate the effect of the groove placing error on the spot profile.

However, for diffraction-limited sources, or, in general, to properly assess the effect of the groove misplacing, it is mandatory to simulate the effect of the groove density errors on the system performance. And here is where *WISER* comes in handy, as will be shown, in detail, in the next section.

## 4. An example: the high-energy-resolution MERLIN beamline at ALS

The MERLIN beamline at the Advanced Light Source (ALS), described by Reininger *et al.* (2007 ▸), is an ultra-high-resolution beamline for the study of low-energy excitations in strongly correlated systems with the use of high-resolution inelastic scattering and angle-resolved photoemission. It covers the energy range 9–150 eV with the target of reaching meV energy resolution [from which MERLIN (‘meV resolution line’) took its name].

A recent project aims to replace the existing monochromator with a Reininger-type monochromator (Reininger & de Castro, 2005 ▸), *e.g.* a variable-included-angle variable-line-space (VLS) grating monochromator, focusing the undulator source directly into the exit slit.

Two plane VLS gratings, named LEG (low-energy grating) and HEG (high-energy grating), are used to cover the energy range in high-resolution mode. A third grating, spherical (*R* = 120 m), with lower groove density, named LRG (low-resolution grating), is used to cover the entire energy range with lower resolution, but higher flux. The source-to-grating distance is 16.58 m and the grating-to-exit-slit distance is 5 m. The gratings parameters are reported in Table 1 ▸.

The effect of the groove placing error for these three gratings has been calculated and simulated considering a fully coherent source. ALS is not a diffraction-limited source and, even when ALS-U is operative, the radiation will be not fully coherent. However, what is important for calculating the effect of the groove misplacing on the grating is the coherent part of the beam. Considering the source to be fully coherent will provide a conservative result, *e.g.* this can be considered the worst-case scenario.

**Figure 7:**
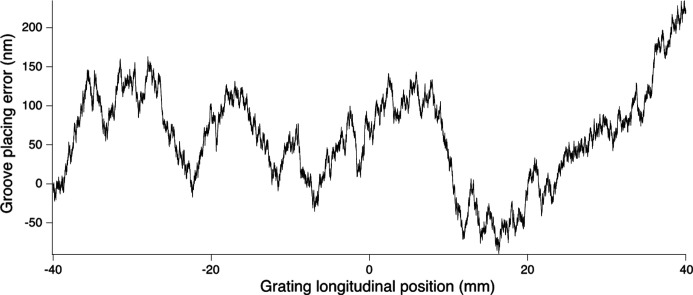
Line error distribution over the central 80 mm of the HEG. The curve represents the distribution for the 62 nm r.m.s. placing error used to simulate the blue curve in Fig. 9 ▸.

To perform the simulation, various groove placing errors have been added to the groove distribution. One example of such a placing error, for the HEG, is shown in Fig. 7 ▸. This error distribution, as well as all the other groove placing errors used, has been generated as Gaussian white noise (with a random seed). The range of used r.m.s. placing errors, for the various gratings, is estimated starting from equation (14). To have enough detail to permit an accurate simulation, the surface describing the gratings contains at least ten points per groove.

**Figure 8:**
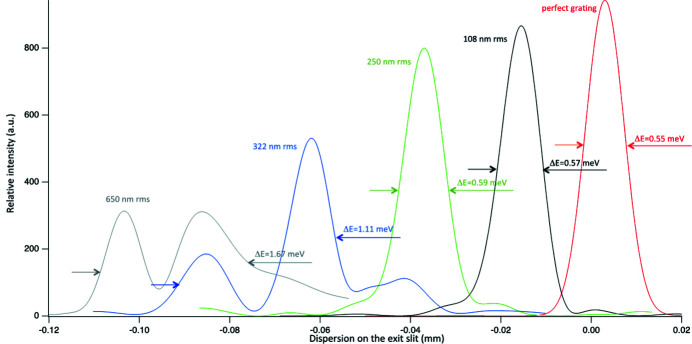
Results of wavefront propagation for the LEG at 30 eV for various r.m.s. groove-placing errors. Each curve is reported with its FWHM in meV and with the associated r.m.s. groove placing error. Line colors match text colors.

Various profiles, corresponding to various groove placing errors, have been created and used for the three gratings described in Table 1 ▸. The results are shown in Figs. 8 ▸, 9 ▸ and 10 ▸. The FWHM bandwidth Δ*E* shown in each plot of Figs. 8 ▸, 9 ▸ and 10 ▸ is calculated from the FWHM spot dimension (*s*) from the relation

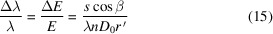

where *r*′ is the distance from the grating to the exit slit. In this particular monochromator, with the target of providing a bandwidth no larger than 1 meV, maximum errors below 200 nm r.m.s. for the LEG and below 40 nm r.m.s. for the HEG are desired. For the LRG, one can relax the requirements to 300 nm r.m.s. (or even more).

If the resolving power is, usually, the most important parameters for a monochromator, the second most important is the flux. The relative flux as a function of the groove placing error should (and can) be calculated using the Strehl ratio as defined in equation (14). For each of the generated groove placing error curves, *S* has been calculated using the r.m.s. groove placing error, calculated over 2 FWHM. The calculated values have been compared with that simulated using *WISER* and are presented in Fig. 11 ▸.

**Figure 10:**
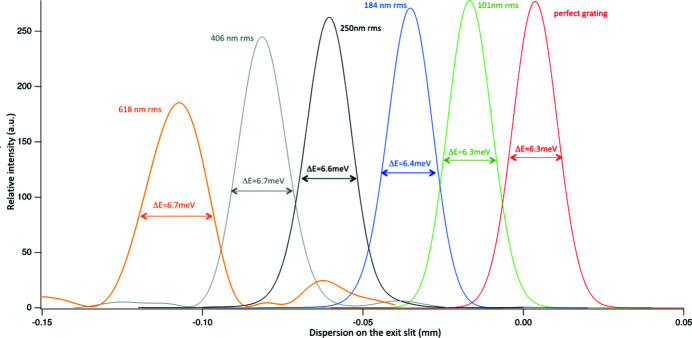
Results of wavefront propagation for the LRG at 80 eV for various r.m.s. groove-placing errors. Each curve is reported with its FWHM in meV and with the associated r.m.s. groove placing error. Line colors match text colors.

**Figure 11:**
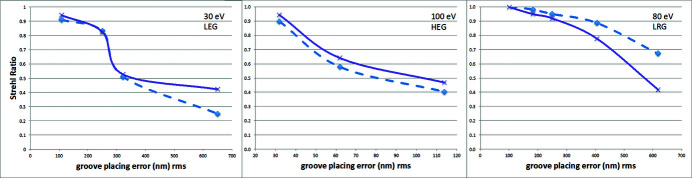
Strehl ratio calculated by using equation (14) (purple lines) over 2 FWHM and simulated using *WISER* (blue dashed lines). The relative intensities are shown in Figs. 8 ▸, 9 ▸ and 10 ▸.

The agreement is satisfactory for medium to high values of *S* (depending on the wavelength), with a discrepancy on the order of few percent when *S* > 0.9. The deviations at small values of *S* (large groove placing errors) are due to the loss of precision of equation (14) when *S* departs too much from unity. In such a case, indeed, the effects of aberrations are not properly considered by this simplified expression. This being cleared up, it is evident that, despite the fact that equation (14) is energy and angle independent, it still represents, reasonably well, the effect of the groove placing errors in terms of intensity reduction. The reduction of energy resolution, directly related to the increase of spot size, is not directly calculable from the Strehl ratio. This is mostly due to the fact that the spot size is affected by several factors. One should deconvolve the effect of all the other causes (including source size, aberrations and so on) to estimate, through a single formula, the effect of the groove misplacement. Moreover, as soon as the spot is no longer Gaussian (as in the case of the HEG in Fig. 10 ▸), the effect on the resolution is more complicated to assess than by simply calculating the ‘width’ of the spot. But there is an interesting coincidence from the three examples shown in this article. In all cases, when *S* < 0.8, the bandwidth increases by more than 10%. This is the usual limit optical designers use to calculate the tolerances of a monochromator. Keeping the Strehl ratio above 0.8 coincides with the Marechal criterion (Marechal, 1947 ▸), for what shall be considered a ‘good’ optical system. As much as we strongly advise performing wavefront propagation simulations to properly assess the effect of the grating’s groove misplacing in a soft X-ray monochromator, the Marechal criterion may be either a good starting point for the simulations or an easy shortcut for avoiding them.

## 5. Conclusions

We have shown how useful, and also how easy, the use of *WISER*, a physical optics simulation package, could be to predict the performance of a grating for coherent sources. It has been shown that the groove placing error, not often properly taken into account while designing a monochromator, can be the most detrimental factor preventing the desired energy resolving power being reached.

## Figures and Tables

**Figure 9:**
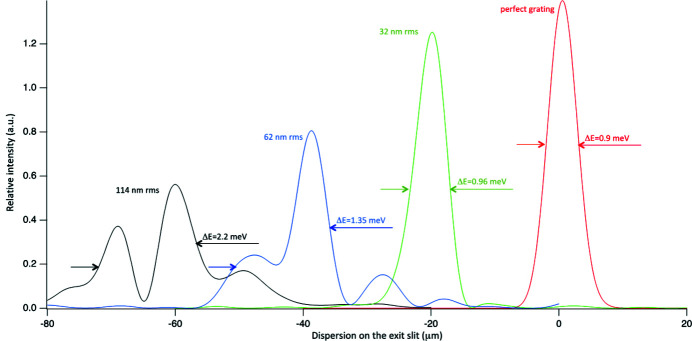
Results of wavefront propagation for the HEG at 100 eV for various r.m.s. groove-placing errors. Each curve is reported with its FWHM in meV and with the associated r.m.s. groove placing error. Line colors match text colors.

**Table 1:** Main parameters of the three gratings of the MERLIN monochromator

Parameter	LEG	HEG	LRG
*D*_0_	600	2400	400
*D*_1_	0.26	1.02	0.2
*D*_2_	7.2 × 10^−5^	0.000282	6.4 × 10^−5^

## Footnotes

1 Available at https://pypi.org/project/LibWiser/.

2 Available at https://pypi.org/project/OASYS1-oasyswiser/ with installation notes for *OASYS*.
